# Supplementary material for: COVID-19 Vaccine Acceptance during Pregnancy and Influencing Factors in South Korea
Source: J Clin Med. 2022 Sep 28;11(19):5733. doi: 10.3390/jcm11195733 (PMC9573627; doi:10.3390/jcm11195733)
Supplement: Supplementary file 1 [file jcm-11-05733-s001.zip › jcm-1911141-supplementary.pdf]

Supplementary Table S1. Geographical distribution of respondents

Distribution	Pregnant or Postpartum Women (n = 436)	
	**N**	**(%)**
Capital region		
Seoul	156	(35.8)
Gyeonggi	122	(28.0)
Non-capital region		
Chungcheong	8	(1.8)
Gyeongsang	19	(4.4)
Jeolla	54	(12.4)
Gangwon	54	(12.4)
Other metropolitan cities (Incheon, Daejeon, Gwangju, Busan, Ulsan, Daegu)	23	(5.3)
